# The psychophysics of sugar concentration discrimination and contrast evaluation in bumblebees

**DOI:** 10.1007/s10071-012-0582-y

**Published:** 2012-11-22

**Authors:** Vladislav Nachev, James D. Thomson, York Winter

**Affiliations:** 1 Department of Biology, Humboldt University, Invalidenstr. 43, 10115 Berlin, Germany; 2 Ecology and Evolutionary Biology Department, University of Toronto, Toronto, Canada

**Keywords:** *Bombus*, Nectarivory, Foraging, Psychometric function, Decision-making

## Abstract

The capacity to discriminate between choice options is crucial for a decision-maker to avoid unprofitable options. The physical properties of rewards are presumed to be represented on context-dependent, nonlinear cognitive scales that may systematically influence reward expectation and thus choice behavior. In this study, we investigated the discrimination performance of free-flying bumblebee workers (*Bombus impatiens*) in a choice between sucrose solutions with different concentrations. We conducted two-alternative free choice experiments on two *B. impatiens* colonies containing some electronically tagged bumblebees foraging at an array of computer-automated artificial flowers that recorded individual choices. We mimicked natural foraging conditions by allowing uncertainty in the probability of reward delivery while maintaining certainty in reward concentration. We used a Bayesian approach to fit psychometric functions, relating the strength of preference for the higher concentration option to the relative intensity of the presented stimuli. Psychometric analysis was performed on visitation data from individually marked bumblebees and pooled data from unmarked individuals. Bumblebees preferred the more concentrated sugar solutions at high stimulus intensities and showed no preference at low stimulus intensities. The obtained psychometric function is consistent with reward evaluation based on perceived concentration contrast between choices. We found no evidence that bumblebees reduce reward expectations upon experiencing non-rewarded visits. We compare psychometric function parameters between the bumblebee *B. impatiens* and the flower bat *Glossophaga commissarisi* and discuss the relevance of psychophysics for pollinator-exerted selection pressures on plants.

## Introduction

Decision-makers such as foraging animals or humans choosing between gambles are able to utilize information about different parameters of the choice options (i.e. probability of reward, amount of reward: Markowitz 1952; Kahneman and Tversky 1979; Wedell 1991; Kacelnik and Brito e Abreu 1998; Bateson et al. 2003; Cnaani et al. 2006; Bacon et al. 2011). Theoretical analyses of choice assume that different reward dimensions are integrated into some common currency, that is, “utility” (Chib et al. 2009; Kenrick et al. 2009). It is further assumed that behaviors maximizing the return currency are associated with fitness benefits and are the products of natural selection (Ritchie 1990; Kenrick et al. 2009). Underlying the capacity to make choices that maximize profitability is the ability to sense and evaluate differences among alternative options (Kacelnik and Brito e Abreu 1998; Livnat and Pippenger 2008; Shafir et al. 2008). Profitability maximization in the case of sequential sampling of multiple options relies on sensation (converting a physical stimulus into a neuronal firing pattern), memory (maintaining a representation of a physical stimulus over a period of time), and decision-making (comparing representations from different sources and performing a motor task based on the results of this comparison). Hereafter, we refer to the conjunction of these three processes as ‘information processing’.

Since the inception of the field of psychophysics, researchers have been interested in the neural and cognitive representations of physical scales (Fechner 1860; Thurstone 1927; Stevens 1961). As direct observations and measurements of subjective sensations are not possible, scientists have instead focused on measuring behavioral output or neuronal activity. Psychometric analyses of scales such as sweetness, heaviness, brightness, and even abstract scales such as time and numerosity typically reveal a nonlinear correspondence between the original scale and the psychological scale (Stevens 1961, 1969; Perez and Waddington 1996; Dehaene 2003; Toelch and Winter 2007; Billock and Tsou 2011; Nachev and Winter 2012). The logarithmic or weak power law compression of sensory information typically observed may result from the tuning properties of sensory neurons (Dayan and Abbott 2001) and has furthermore been suggested not only for sensory traces, but also for reactivated memories as well (Gallistel and Gelman 2000; Nieder and Miller 2003; Papini and Pellegrini 2006). This type of representational mechanism is robust against errors and arguably superior to alternative mechanisms (Sinn 2003; Portugal and Svaiter 2010), but it can influence choice behavior in a systematic way (Livnat and Pippenger 2008; Nachev and Winter 2012). For example, in a choice between two alternative magnitudes (e.g. numbers, sucrose concentrations, or volumes), discrimination performance is expected to improve as the difference between the options increases (distance effect) and decline as distance (the absolute difference between the two options) is kept constant but the average magnitude of the two options increases (magnitude effect, a consequence of the nonlinear compression of sensory information).

A well-established tradition uses honeybees (Apinae: Apini) and more recently bumblebees (Apinae: Bombini) as model organisms for studying foraging behavior and decision-making (von Frisch 1927; Real 1981; Schmid-Hempel 1987; Schmid-Hempel and Schmid-Hempel 1987; Harder 1988; Waddington and Gottlieb 1990; Shafir et al. 2002, 2008; Heinrich 2004; Waldron et al. 2005; Cnaani et al. 2006; Gil 2010). However, despite the investigations into the mechanisms of information processing in these insects (Waddington and Gottlieb 1990; Shafir 2000; Waddington 2001; Shafir et al. 2002, 2008; Waldron et al. 2005; Gil 2010), the relationship between information processing and choice profitability remains unclear. It has been demonstrated that bees form reward expectations (Gil 2010) and it has been suggested that the differences between the expectation and the actual perceived reward shape the development of economic flower preferences (Waldron et al. 2005; Wiegmann and Smith 2009). An important question that still needs to be addressed is how well bees track differences along reward dimensions while foraging under conditions similar to the natural situation, where there is uncertainty whether a flower contains any nectar.

In this study, we investigated the ability of the Common Eastern Bumblebee *Bombus impatiens* to discriminate between sucrose solutions with different sugar concentrations. Previous experiments have already shown that bumblebees are very sensitive to differences in sucrose concentration (Waddington 2001; Waldron et al. 2005; Cnaani et al. 2006; Wiegmann and Smith 2009). These studies suggest a nonlinear relationship between objective sucrose concentration (weight/weight percentage) and subjective evaluation (Waddington 2001) and indicate that foraging choices do not always conform to predictions based on net energy gain maximization (Schmid-Hempel 1987; Waldron et al. 2005; Cnaani et al. 2006). However, the precise functional relationship between discrimination performance and concentration has not yet been investigated.

A traditional psychophysical method for estimating discrimination performance is fitting a psychometric function to data from n-alternative force choice tasks (*n*-AFC: Treutwein and Strasburger 1999). The psychometric function takes a measure of the intensities of the presented stimuli as argument and gives the discrimination performance, for example, the probability with which an observer judges one stimulus to be larger in magnitude from another stimulus. In previous two-alternative choice experiments with nectar-feeding bats (Toelch and Winter 2007; Nachev and Winter 2012), the ratio of the linear difference of the stimuli to the average stimulus value was proposed as the appropriate intensity measure, because it captures the expectations that discrimination performance should increase with the difference (distance effect) and decrease with the mean magnitude of the two options (magnitude effect).

The psychometric functions are typically assumed to have a sigmoidal shape and are modeled as the distribution functions of the normal, logistic, Weibull, or Gumbel distributions (Treutwein and Strasburger 1999; Kuss et al. 2005). Parameterization of the functions is preferably made so that the parameters have a meaningful biological interpretation, as is the case with the Weibull parameterization (Kuss et al. 2005; Fründ et al. 2011). The three parameters in the Weibull parameterization are the threshold, slope, and lapse rate. The threshold is the point on the curve that is halfway between the lower and the upper asymptote. In 2-AFC experiments, it usually corresponds to a discrimination performance around 75 %. The slope of the function is measured at the threshold and has been proposed as a reliability measure of sensory performance (Strasburger 2001). Finally, the lapse rate is seen as a measure of the frequency of errors due to motivational problems and other factors of non-perceptual nature. The lapse rate is a measure that depends on the particular task given and we suggest that in animal studies, lapsing can also result from exploratory behavior (or from competition avoidance). Foraging animals face the exploration–exploitation dilemma and will not necessarily always make choices based on expected values. In psychometric analyses, it is assumed that a forager has a constant lapse rate, that is, a constant probability to select an option not based on stimulus intensity. When a forager lapses during a specific choice in a 2-AFC experiment, its probability of selecting the correct option is at the chance level of 0.5 and equals the probability of selecting the incorrect option. Therefore, the lapse rate is calculated as one minus the upper asymptote of the psychometric curve (the estimated base rate of incorrect choices) multiplied by two.

To the best of our knowledge, a psychometric function for sugar concentration discrimination performance has so far only been fitted for one species, the nectar-feeding bat *Glossophaga commissarisi* (Nachev and Winter 2012). The estimates for the lapse rate, threshold, and slope were 0.04, 0.50, and 3.41, respectively. In a recent dynamic modeling study of nectar extraction, the optimal sugar concentration for viscous dippers (animals that extract flower nectar by repeatedly dipping and retracting their tongues in the viscous liquid) was estimated at 52 % w/w (Kim et al. 2011). However, although both bumblebees and bats are classified as viscous dippers (Kim et al. 2011), typical bat-pollinated plants have nectars with much lower sugar concentrations (13–18 % w/w: Pyke and Waser 1981; von Helversen and Reyer 1984) than typical bee-pollinated plants (35 % w/w: Pyke and Waser 1981). This difference cannot be explained by differences in nectar-drinking style as modeled by Kim et al. (2011). On the other hand, differences in discrimination performance between the two groups of pollinators might influence the evolution of nectar concentrations in the plants they pollinate. Since bumblebees live in an ecological environment with higher nectar sugar concentrations than flower bats, bumblebees may be expected to have a better developed ability for concentration discrimination. This is because of the magnitude effect. At the higher end of a perceptive scale, that is, a higher sugar concentration, a higher sensitivity is required to discriminate between options that differ by a given distance in stimulus intensity. Here, we present the first psychometric analysis of sugar concentration discrimination performance in a nectar-feeding insect, based on two-alternative, free choice experiments with individually identifiable *B. impatiens* workers foraging on an array of computer-automated artificial flowers.

## Methods

### Bumblebees

We worked consecutively with two bumblebee colonies initially containing about 20–30 workers (Colony 1) and 40 workers (Colony 2) of *B. impatiens* (BioBest Canada Ltd, Leamington, ON, Canada). The experiments were carried out at the University of Toronto, Ontario, Canada. Nest boxes (29 × 21 × 14 cm) were connected by tunnels to a training cage (77 × 76 × 79 cm) where two artificial flowers (see below) provided nectar (sucrose aqueous solution, 20 % w/w). After a training period of 6 days, the nest box was connected to one of the long walls of the test cage (293 × 245 × 219 cm) inside the same room. The test cage was equipped with six fluorescent lights providing a mixture of ultraviolet and white light. These lights were kept on a LD 12:12 schedule, while dimmed fluorescent white lights higher above the cage were kept on continuously. Commercial pollen was supplied as a food supplement directly to the colonies on a daily basis. We captured 75 foraging individuals and marked them with unique radiofrequency identification tags (RFID, PhenoSys, Germany). The tags were glued on the scuta of cold-anaesthetized bumblebees with cyanoacrylate glue (Instant KrazyGlue Gel Formula, Columbus, OH, USA). Bumblebees were then released in the test cage, where they could resume foraging.

### Artificial flowers

**Fig. 1:**
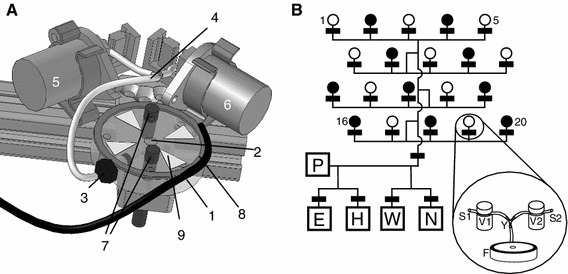
Artificial bumblebee flowers. **a** Schematic view of a single flower. Bumblebees land on an acrylic platform (*1*) and collect nectar from the nectar bucket (*2*). The bucket is filled through a horizontal hole connected via a thread-to-barb connector (*3*) to a nectar tube (*4*). Direct access to the nectar in the horizontal hole is prevented by a plastic baffle (not shown here, see Ohashi et al. 2010 for details). The tube receives nectar from either one of the two pumping systems, the tubes of which merge with (*4*). Nectar supplied from the two systems is directed to *4* by pinch valves (*5* and *6*). The delivery of nectar can be triggered when the infrared sensor’s (*7*) light beam is interrupted. If the bumblebee carries a tag, its unique number is detected by an antenna (*8*) and sent to the transponder reading device (not shown). Flower nectar quality is indicated with *color* cues: triangular ‘petals’ cut from electrical tape (*9*) and adhered to (*1*). **b** Pump and tubing system of the 20-feeder array. *Lines* represent the tubes, and *black rectangles* the pinch valves. Feeders are numbered *1*–*20*. *Boxes* represent the following liquid reservoirs: ethanol (*E*), water (*H*), waste (*W*), nectar (*N*), stepping-motor syringe pump (*P*), as described in Winter and Stich (2005). Length of tubes not drawn to scale. Two identical tubing systems were connected to the feeders. The merging point of the tubing systems is illustrated in the *inset*: magnetic pinch valves for the first (*V1*) and second (*V2*) tubing systems with their corresponding tubes (*S1* and *S2*), a *Y* connector (*Y*), and feeder platform (*F*). Feeders shown in *black* had blue ‘petals’ and only received nectar from *S1*, and feeders shown in *white* had yellow ‘petals’ and only received nectar from *S2*. The two pumping systems were filled with different sugar concentrations on different days. See “Methods” for details

Visits to the artificial flowers or feeders (PhenoSys, Germany) were registered with an infrared sensor (Fig. 1). Transponder reading devices identified individuals carrying radiofrequency identification (RFID) tags. Each feeder was equipped with two solenoid pinch valves that controlled nectar delivery via two tubing systems (Fig. 1). Nectar rewards were delivered to a nectar bucket inside the feeder platform, a vertical hole with 5 mm diameter and 7 mm depth. The design of the nectar bucket was made after Ohashi et al. (2010) and included a plastic baffle to prevent bumblebees from getting nectar directly from the incoming tube (Fig. 1a). Nectar volume and concentration were controlled by two syringe pumps (PhenoSys, Germany) using two gas-tight Hamilton glass syringes (Series 1002, total volume 2.5 ml). After delivering a 5-μl reward, a feeder became unrewarding for 10 s, as an incentive for bumblebees to search for nectar elsewhere rather than collect multiple rewards at the same feeder. We assumed that bumblebees collected the full reward volume on every visit. If a bumblebee obtained a reward at a feeder and remained on it for longer than 10 s, it would need to leave the receptive field of the feeder’s sensors in order to terminate the visitation event, before a further reward could be delivered. As bumblebees foraged simultaneously, the probability that a feeder would be unrewarding depended on the activity of the foragers, a situation that mimics natural foraging conditions. In order to make feeders more conspicuous and to promote learning, we adhered triangular ‘petals’ made from colored electrical tape to the feeder platforms. We used red and white tapes for the two training feeders and blue and yellow tapes for the feeders in the main experiment.

For the main experiment, we used a staggered 4 × 5 array of twenty computer-controlled feeders (Fig. 1b). Feeders were mounted on inverted flower pots, positioning the top of the landing platform approximately 10 cm above the floor. Feeders were spaced 40 cm apart. The whole array was positioned on the floor inside the test cage, about 50 cm from the two short walls and the long wall opposite the entrance point. The control computer, hardware interface, power supply units, and nectar reservoirs were all placed on a laboratory cart outside the back of the cage and connected to the feeders via signal cables and main nectar tubes. One pumping system supplied the blue-petaled feeders, the other the yellow. The two systems were filled with nectars with different concentrations. Thus, during a single experimental session, the concentration offered at each feeder was fixed and did not change. In order to prevent bacterial and fungal growth inside the tubing systems, they were rinsed with water and a 70 % ethanol solution every 3–4 days.

### Experimental procedure

Bumblebees were first trained on the two feeders inside the training cage for 6 days, and then their nest box was connected to the experimental cage. The tunnel that connected the colony to the cage was kept closed during the ‘dark’ phase and opened within 1–2 h after the start of the ‘light’ phase. On the first experimental day for each colony, the entrance to the cage was smeared with honey, as an incentive for bumblebees to explore the cage. On the following days, bumblebees spontaneously left their nest box as soon as the connecting tunnel was opened. A foraging session began with the opening of the connecting tunnel and ended 12 h later, when reward delivery at the feeder array was automatically stopped. Most bumblebees would then spontaneously return to their nest box. The remaining individuals were netted and placed in the nest box. All feeder visits during a foraging session were recorded.

We chose concentrations from the natural range of floral nectars (Pyke and Waser 1981), ranging from 15 to 50 % sucrose/water weight/weight (or 464–1,796 mmol l^−1^, Bolten et al. 1979). For each of the two colonies, we conducted a series of two-alternative free choice tests, with 10 feeders per option (Table 1). Every concentration pair was presented twice on consecutive days, with the positions of the two concentrations exchanged as a control for positional and color biases (Fig. 1b). This resulted in reversal test conditions for the bumblebees on nearly every day (Table 1). All experiments were performed with PhenoSys (Germany) experimental control software.

**Table 1:** Discrimination performance (response) for different sucrose concentrations in *B. impatiens* workers from two different colonies

Days	Blue^a^	Yellow^a^	Average^a^	Intensity^b^	*N* bees^c^	*N* visits × 1,000^d^	Response^e^
*Colony 1*							
1	30	15	22.5	0.67	0 (0)	– (–)	– (–)
2	15	30	22.5	0.67	1 (5)	0.59 (0.90)	0.96 (0.98)
3	45	30	37.5	0.40	1 (4)	1.04 (0.76)	0.87 (0.94)
4	30	45	37.5	0.40	0 (6)	– (0.77)	– (0.95)
5	30	20	25.0	0.40	0 (7)	– (0.94)	– (0.87)
6	20	30	25.0	0.40	1 (9)	0.02 (1.52)	0.92 (0.88)
7	45	50	47.5	0.11	1 (8)	1.90 (1.71)	0.71 (0.7)
8	50	45	47.5	0.11	0 (9)	– (1.82)	– (0.62)
9	35	45	40.0	0.25	0 (6)	– (0.86)	– (0.79)
10	45	35	40.0	0.25	1 (9)	1.20 (1.85)	0.83 (0.8)
11	30	32	31.0	0.06	1 (7)	0.11 (1.62)	0.45 (0.48)
12	32	30	31.0	0.06	1 (9)	0.44 (1.50)	0.49 (0.46)
13	40	20	30.0	0.67	2 (13)	3.01 (2.39)	0.89 (0.89)
14	20	40	30.0	0.67	2 (12)	4.45 (1.98)	0.92 (0.9)
15	35	39	37.0	0.11	2 (14)	1.49 (2.56)	0.83 (0.67)
16	39	35	37.0	0.11	1 (17)	0.47 (3.12)	0.56 (0.63)
*Colony 2*							
1	50	45	47.5	0.11	5 (20)	3.35 (5.01)	0.52 (0.57)
2	45	50	47.5	0.11	2 (10)	0.06 (2.40)	0.52 (0.57)
3	45	30	37.5	0.40	0 (11)	– (2.73)	– (0.85)
4	30	45	37.5	0.40	4 (9)	2.71 (2.13)	0.96 (0.92)
5	25	20	22.5	0.22	3 (9)	1.20 (2.43)	0.54 (0.65)
6	20	25	22.5	0.22	4 (11)	5.51 (2.40)	0.6 (0.62)
7	30	15	22.5	0.67	4 (12)	5.64 (2.94)	0.87 (0.84)
8	15	30	22.5	0.67	5 (12)	5.31 (3.47)	0.88 (0.79)
9	34	25	29.5	0.31	3 (12)	2.65 (2.89)	0.93 (0.91)
10	25	34	29.5	0.31	3 (16)	2.92 (3.53)	0.83 (0.66)
11	27	21	24.0	0.25	4 (16)	4.36 (3.50)	0.58 (0.56)
12	21	27	24.0	0.25	3 (14)	3.16 (3.08)	0.72 (0.68)

Bees were presented with 20 artificial flowers with blue (*N* = 10) and yellow petals (*N* = 10), and the relative preference for the feeders with the sweeter nectar was calculated for bumblebees that made at least 800 visits

^a^Sucrose solution concentrations are given in % weight/weight

^b^Relative intensity is calculated as the difference between the two concentrations (blue and yellow) divided by the average of the concentrations

^c^Numbers without parentheses give the number of marked bumblebees that made at least 800 visits. Numbers in parentheses give the number of unmarked bumblebees, estimated by dividing the total number of recorded unidentified visits for that day by the average number of visits per day for unmarked bumblebees over the whole experiment of the respective colony

^d^Numbers without parentheses give the total number of visits (in thousands) made by marked bumblebees, excluding the first 800 visits per individual. Numbers in parentheses give the total number of visits (in thousands) made by unmarked bumblebees, excluding the first *m* visits, where *m* is 800 × estimated number of unmarked individuals

^e^Numbers without parentheses give the weighted average response of marked bumblebees using the individual number of visits as weights. Numbers inside parentheses give the response of unmarked bumblebees. The marked and unmarked bumblebee responses were positively correlated in Colony 1 (Spearman’s rank correlation = 0.92, *S* = 18, *p* < 0.001, *N* = 11 days) and in Colony 2 (Spearman’s rank correlation = 0.91, *S* = 20.55, *p* < 0.001, *N* = 11 days)

## Data analysis

Recorded data comprised the time-stamped visitation events of marked and identified and of unmarked bumblebees. In order to focus on the plateau performance of bees that had become familiar with the choices being offered, after the initial sampling and exploration phase, we excluded the first 800 visits from the analysis of marked bumblebee data. Visual inspection of the daily learning curves confirmed that no substantial changes in feeder preference occurred after the 800-visit cut-off point. A total of 34 marked bumblebees made at least 50 visits on at least 1 day and 13 marked bumblebees made at least 800 visits on at least 1 day. Out of these individuals, three bumblebees retained their transponders for a sufficient number of days and made a sufficient number of visits to permit individual-based psychometric analyses for these three animals. The three individuals came from the second colony. Otherwise, we analyzed unmarked bumblebee data collectively. We estimated the number of visits per bumblebee by taking the recorded mean daily visits by the 34 marked bumblebees that made at least 50 visits on at least 1 day. We then estimated the number of foraging individuals by dividing the total number of unmarked visits by the estimate for the number of visits per bumblebee. For the asymptotic performance of the unmarked bumblebees, we assumed the same cut-off point of 800 visits per bee and approximated it by excluding the first *m* visits, where *m* was 800 multiplied by the estimated number of unmarked individuals. For each marked bumblebee, and for the unmarked bumblebees from each colony, we calculated the *relative intensities* (treatment) and the *discrimination performances* (response) for each experimental day. The *relative intensity* was calculated as the absolute difference between the two sucrose concentrations expressed in percentage weight/weight, divided by the mean concentration. Here, we adopt this measure on theoretical grounds (Toelch and Winter 2007; Nachev and Winter 2012) without explicitly testing the separate contributions of the distance and magnitude effects. The response was calculated as the number of visits to higher concentration feeders divided by the total number of visits. We calculated separate responses for each day; for further analyses, we combined the daily responses as the weighted average over the two presentations of the same condition, using number of registered visits as weights. This step was intended to control for positional or color biases. Statistical analysis was carried out using R 2.10.1 (R Development Core Team 2009).

### Psychometric analysis

We performed psychometric analyses on the response data from each animal and each colony (unmarked bumblebees) and fitted Weibull psychometric functions using the algorithm proposed by Kuss et al. (2005) with *relative intensity* as independent and *discrimination performance* as dependent variables (Toelch and Winter 2007; Nachev and Winter 2012). This Bayesian approach yields estimates for the threshold, slope, and lapse rate of the psychometric function, as well as confidence intervals for these parameters, using Markov Chain Monte Carlo (MCMC) sampling. For the threshold, we chose a normally distributed prior with a mean of 1 and a standard deviation of 0.5, and for the slope, a normal prior with a mean of 2 and a standard deviation of 1. In human experiments, the lapse rate is usually in the range 0.01–0.10, but instead of restricting the prior to this range, we selected as prior the beta distribution (2;20), in order to allow for higher lapse rates due to exploratory behavior. We performed 5,000 MCMC sampling runs with a leapfrog step size of 100 to obtain the mean and 95 % confidence intervals for the threshold, slope, and lapse rate.

## Results

**Fig. 2:**
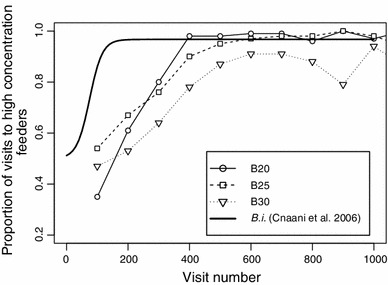
Learning curves for *B. impatiens* in sucrose discrimination tasks. *Open symbols* give the proportion of visits to the higher concentration feeders calculated over bins of 100 visits. For each of the three focus individuals from this study, the steepest learning curves from the complete data sets were selected. For B20, B25, and B30, data were taken from the first 1,000 visits on days 4, 8, and 7, respectively. The *thick line* is calculated from the learning curve parameters estimated in a previous study with *B. impatiens* (Cnaani et al. 2006: Table 2, ‘Concentration 30’). The concentrations used in that study were 13 and 40 % (weight/weight)

**Fig. 3:**
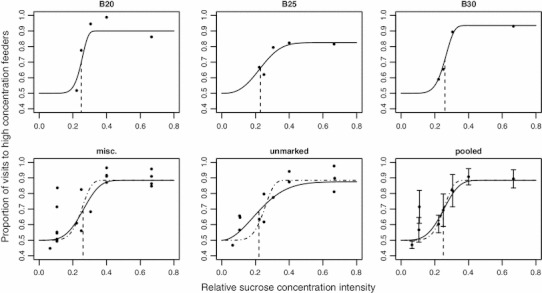
Psychometric curves for sucrose concentration discrimination. Sucrose concentration intensities are given on the abscissa and are calculated as the absolute value of the difference divided by the mean of two concentrations (see “Methods”). *Black circles* represent weighted average responses (proportion of visits to the higher sucrose concentration) over two presentations of the same pair of sucrose concentrations (Table 1), using number of visits as weights. The continuous curves represent the respective psychometric functions, and the *dashed vertical lines* indicate the psychometric function thresholds. The top three panels from *left* to *right* give data from three individually marked bumblebees. The *bottom left panel* gives the weighted average responses of marked bumblebees from both colonies that satisfied the minimum 800 visits per day criterion, but were not detected on a sufficient number of days for individual psychometric analysis. (Most of these data points are for single days only, rather than average values over 2 days.) The *bottom middle panel* gives the weighted average responses of all unmarked bumblebees from both colonies, and the *bottom right panel* gives the average responses (*circles*) and standard deviations (*whiskers*) calculated from pooling all data together (B20, B25, B30, miscellaneous, unmarked). The *dashed curves* in the *bottom panels* represent the psychometric function with parameters (lapse rate, threshold, and slope) averaged over the parameters of the three individually marked bumblebees

On average, each of the 34 marked bumblebees made 1,076 ± 642 visits bee^−1^ day^−1^ (mean ± SD, excluding individuals which made fewer than 50 visits, *N* = 27 days) and the total of unmarked bumblebees made 10,754 ± 4,065 visits day^−1^ (mean ± SD, *N* = 27 days). Even after the first 800 visits, marked bumblebees usually continued to visit almost all of the 20 available feeders (mean ± SD = 18.5 ± 1.63 feeders, *N* = 15 bumblebees) thus visiting both concentration types. However, they seldom distributed their visits evenly among the feeders. Even at the highest stimulus intensities, bumblebees made at least 400–600 visits before reaching asymptotic performance in their choice behavior (Fig. 2). They showed no discrimination between concentrations at low relative intensities and good discrimination at high relative intensities. This led to psychometric functions that are nonlinear for the variables we have chosen (Fig. 3).

In Colony 1, the proportion of non-rewarded visits (visits within the 10-s refill delay) at feeders with lower concentration (mean ± SD = 0.47 ± 0.10, *N* = 15 days) was the same as at feeders with higher concentration (mean ± SD = 0.48 ± 0.06, *N* = 15 days; paired t test: *t*(14) = −0.64, *p* = 0.53). In Colony 2, the feeders with lower concentrations had a lower frequency of non-rewarded visits (mean ± SD = 0.54 ± 0.03, *N* = 12 days) than feeders with higher concentrations (mean ± SD = 0.58 ± 0.03, *N* = 12 days; paired t test: *t*(11) = −5.39, *p* < 0.001), but the difference was small.

As seen in Table 2, the psychometric function thresholds estimated from individually analyzed marked bumblebees (mean ± SD = 0.25 ± 0.01, *N* = 3 bumblebees) were similar to the values obtained from pooling miscellaneous marked bumblebees (0.24) and similar to the values from all unmarked bumblebees (0.22). The individually estimated lapse rates (mean ± SD = 0.23 ± 0.11, *N* = 3 bumblebees) were also similar to the estimates obtained from pooling miscellaneous marked bumblebees (0.18) and all unmarked bumblebees (0.25). Finally, the psychometric function slopes varied strongly from individual to individual (mean ± SD = 8.22 ± 3.80, *N* = 3 bumblebees), and the corresponding estimates for miscellaneous marked individuals and for all unmarked individuals were lower, at 3.29 and 3.12, respectively (Table 2).

**Table 2:** Psychometric function parameters for discrimination of sucrose solution concentrations in *B. impatiens* workers

Bumblebee	Lapse rate^a^	Threshold^a^	Slope^a^	*N* (days)
B20	0.19 | 0.20 | 0.21	0.244 | 0.247 | 0.25	10.80 | 11.67 | 12.59	7
B25	0.33 | 0.35 | 0.38	0.22 | 0.23 | 0.24	3.19 | 4.15 | 5.08	9
B30	0.12 | 0.13 | 0.14	0.256 | 0.26 | 0.263	8.05 | 8.82 | 9.62	7
Misc.^b^	0.17 | 0.18 | 0.19	0.23 | 0.24 | 0.26	2.68 | 3.29 | 4.35	24
Unmarked^c^	0.24 | 0.25 | 0.26	0.21 | 0.22 | 0.23	2.95 | 3.12 | 3.29	27
Pooled^d^	0.22 | 0.23 | 0.23	0.248 | 0.251 | 0.253	4.80 | 5.30 | 5.80	27

^a^Parameters estimated with a Bayesian Markov Chain Monte Carlo (MCMC) sampling method (Kuss et al. 2005). Values in the middle are average estimates, and the values to the left and right are the 95 % confidence interval limits

^b^Analysis based on pooled data from miscellaneous marked bumblebees that made sufficient number of visits on some days, but were not detected over a sufficient number of days for individual psychometric analysis (*N* = 10 bumblebees)

^c^Analysis based on data from all unmarked bumblebees

^d^Analysis based on pooled data from all bumblebees (B20, B25, B30, misc., and unmarked)

## Discussion

Our bumblebees could choose between two types of sugar solutions that differed on different experimental days in their relative intensity to each other. Depending on relative intensity of difference between options, *B. impatiens* workers were either indifferent to differences in sucrose concentration or made more visits to the feeders with the higher concentration. Their discrimination performance can be described by the psychometric function presented in this study (Fig. 3). In general, the predicted relative visitation rate to the sweeter option of two concentrations (from the range 15–50 % w/w) with relative intensity *x* can be calculated with the following equation:

1 $$ \Uppsi (x,m,s,\pi_{l} ) = \frac{1}{2}\left[ {\pi_{l} + (1 - \pi_{l} )\left[ {2 - \exp \left( { - \exp \left( {\frac{2sm}{\ln (2)}(\ln (x) - \ln (m)) + \ln (\ln (2))} \right)} \right)} \right]} \right] $$

where *m* is the threshold, *s* is the slope at the threshold, and *π*_*l*_ is the lapse rate (from equations (1) and (11) in Kuss et al. 2005). For instance, the psychometric function predicts that for intensities higher than the threshold (*x* > 0.25, Table 2), the options with the more concentrated nectars will receive at least 70 % of all visits. Because of the somewhat high estimated lapse rates (Fig. 3; Table 2), the psychometric function likely underestimates the perceptual capacity for sugar discrimination in bumblebees. Caution should also be taken when using concentrations higher than 50 % w/w, as viscosity and extraction costs are known to increase with concentration (Harder 1986; Kim et al. 2011) and may invalidate predictions based on the psychometric function. Whether that is the case could be tested by disassociating viscosity from sweetness using the inert polymer Tylose (Josens and Farina 2001; Borrell 2006; Köhler et al. 2010).

**Fig. 4:**
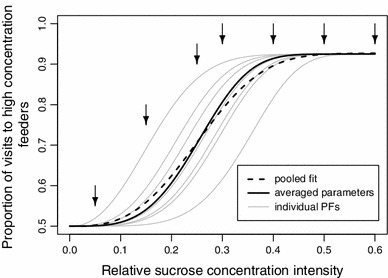
Data pooling can cause underestimation of the psychometric function slope. The figure illustrates with a theoretical example how the averaging of individual data changes psychometric function parameters. We start with 7 “individuals” represented by psychometric functions (PFs, *gray lines*) with different thresholds (mean ± SD: 0.25 ± 0.057), but equal lapse rates (0.15) and slopes (5). From the individual curves, we calculate the predicted discrimination performance values at relative intensities 0.05, 0.15, 0.25, 0.3, 0.4, 0.5, and 0.6 (*arrows*). We then average the predicted discrimination performances across animals using 200 visits per animal for each intensity value (*N* = 200 visits × 7 animals = 1,400 visits per relative intensity value) and apply the algorithm for psychometric function fitting by Kuss et al. (2005). We use a flat prior for the slope, in order to exclude potential confounding effects of the prior and select all remaining parameters as described in the “Methods” section. The resulting psychometric curve (*dashed line*) has a slope (±95 % CI) of 4.07 ± 0.67, significantly lower than the actual value of 5 that was identical for all individuals in the initial theoretical functions (*p* < 0.05). The estimates for the lapse rate (0.15 ± 0.02) and threshold (0.25 ± 0.01) do not differ from the average parameters. For comparison, the psychometric curve with parameters averaged across animals is also shown (*continuous black line*)

When comparing the individually calculated psychometric functions with functions fitted on pooled data from unmarked or miscellaneous marked bumblebees (Fig. 3; Table 2), the different data sets yield similar estimates for the threshold (all in the range 0.22–0.26) and are consistent with respect to the lapse rate (all in the range 0.18–0.25). As shown in the results and in Fig. 4, the slope is underestimated when pooled data from unmarked or miscellaneous marked bumblebees are analyzed instead of separately analyzing individual data. We conclude from this that if researchers are primarily interested in estimating the threshold rather than the slope, then similar psychometric studies (e.g. on nectar volume, or probability of reward) can be conducted without the individual transponder tracking used in this study.

Gustatory perception of sucrose concentration depends on chemoreceptors on bees’ glossae (Whitehead and Larsen 1976), and evaluation of this information is probably immediate. Yet bumblebees needed several hundred visits to reach asymptotic performance in their choice behavior (Fig. 2). The lower learning rates in comparison with the rates reported by Cnaani et al. (2006) may possibly reflect the difficulty of performing a spatial reversal task in our experiments. We interchanged the positions of higher and lower quality feeders in the experimental array daily. Impeded learning could also be explained by differences in salience of the sensory cues (visual vs. olfactory) or by a possible confounding effect of the 10-s delay rule (see “Methods”), which led to ca. 50 % unrewarded visits.

The psychometric function predicts that bumblebee workers will be indifferent to sugar concentration differences below a relative intensity value of about 0.1. However, strong preferences for one feeder type over the other were detected in some marked bees even below this value (Table 1, Colony 1, days 7 and 15; see also Fig. 3, bottom left panel, points at 0.11 relative intensity). This discrimination performance may have been facilitated by a carryover effect from the previous day providing a learning phase with 2-day duration. On experimental days 7 and 15, in deviation from regular routine, there was no reversal with respect to the previous days, that is, the higher concentrations were in the same colored feeders for two consecutive nights (Table 1). It appears that in the absence of strong sugar concentration differences, some bumblebees did not update the remembered value of the lower concentration type as fast as others.

It has been hypothesized that the difference between reward expectation and actual perceived reward drives the choice for more profitable food options in bees (Waldron et al. 2005; Wiegmann and Smith 2009). There is some field evidence that bumblebees employ a win-stay, lose-shift strategy: when they consecutively experienced low reward volumes (estimated by measuring flower handling time as proxy) at one flower species, they were more likely to switch to another species (Chittka et al. 1997; but see Bar-Shai et al. 2011). In addition to the difference between the two sucrose concentrations, the bumblebees in our experiment could also experience unrealized reward expectations when making a non-rewarded visit at each feeder type. One way to demonstrate a negative incentive contrast of this kind is to show that after experiencing two unrewarded visits at high concentration feeders (e.g. blue), bumblebees are more likely to sample a low concentration feeder (e.g. yellow) than after experiencing a reward followed by a non-rewarded visit at blue feeders (Prediction 1). (Hereafter, we refer to the high concentration feeders as blue and low concentration feeders as yellow for ease of explanation). Similarly, if the remembered value of a feeder is downgraded after a non-rewarded visit, then bumblebees should be more likely to sample a yellow feeder after making two unrewarded visits at blue feeders than after making two rewarded visits at blue feeders (Prediction 2). In order to test these predictions, we looked at the first 800 visits marked bumblebees made on days with relative intensity of 0.67 (the condition with the highest number of detected marked bumblebees). We excluded animals if they did not develop a preference above 90 % for blue feeders and performed paired t tests with probability to shift from blue to yellow as the dependent variable and the last two reward experiences (two rewards, or one reward followed by no reward, or two unrewarded visits) as the independent variable. Our results failed to support Prediction 1 (paired *t*(6) = −1.989, *p* = 0.09, *N* = 7 bumblebees) and Hypothesis 2 (paired *t*(6) = −2.454, *p* = 0.0495, *N* = 7 bumblebees). In both cases, the differences were in the opposite direction of the predicted, that is, bumblebees were more likely to shift to yellow after experiencing two rewards at blue feeders than after experiencing two non-rewarded visits at blue feeders. Our interpretation of these results is that bumblebees do not update the expected value of color marked feeders when experiencing non-rewarded visits.

Despite the uncertainty and frequent changes in feeder quality, the psychometric function that describes the discrimination performance of *B. impatiens* workers is finely tuned, with a lower threshold (0.25) and a steeper slope (5.3) than the mean threshold (0.50) and slope (3.3) of *G. commissarisi* bats measured in a similar two-alternative free choice task (Nachev and Winter 2012). In other words, bumblebees seem to be better at discriminating small differences between sugar concentrations than nectar-feeding bats. As described in the introduction, bumblebee-pollinated plants have on average sweeter nectars than bat-pollinated plants. Here, we show that the groups also differ in their psychometric functions of sweetness perception. This raises the question how the evolution of plant nectar traits and pollinator information-processing mechanisms might be related.
